# Random Lasers Based on Tellurite and Germanate Glasses and Glass-Ceramics Doped with Rare-Earth Ions

**DOI:** 10.3390/mi16050550

**Published:** 2025-04-30

**Authors:** Davinson M. da Silva, Josivanir G. Câmara, Niklaus U. Wetter, Jessica Dipold, Luciana R. P. Kassab, Cid B. de Araújo

**Affiliations:** 1 Faculdade de Tecnologia de São Paulo, Pça Cel. Fernando Prestes, 30, São Paulo 01124-060, SP, Brazil; kassablm@osite.com.br; 2 Escola Politécnica, Universidade de São Paulo, São Paulo 05508-970, SP, Brazil; josivanir@usp.br; 3 Instituto de Pesquisas Energéticas e Nucleares, IPEN/CNEN, Cidade Universitaria—CEP, Av. Prof. Lineu Prestes, 2242, São Paulo 05508-000, SP, Braziljessica.dipold@gmail.com (J.D.); 4 Departamento de Física, Universidade Federal de Pernambuco, Recife 50670-901, PE, Brazil

**Keywords:** random lasers, glasses, glass-ceramics, upconversion, replica symmetry breaking

## Abstract

Random lasers (RLs) based on glasses and glass-ceramics doped with rare-earth ions (REI) deserve great attention because of their specific physical properties such as large thermal stability, possibility to operate at high intensities, optical wavelength tunability, and prospects to operate Fiber-RLs, among other characteristics of interest for photonic applications. In this article, we present a brief review of experiments with RLs based on tellurite and germanate glasses and glass-ceramics doped with neodymium (Nd³⁺), erbium (Er³⁺), and ytterbium (Yb³⁺) ions. The glass samples were fabricated using the melt-quenching technique followed by controlled crystallization to achieve the glass-ceramics. Afterwards, the samples were crushed to obtain the powder samples for the RLs experiments. The experiments demonstrated RLs emissions at various wavelengths, with feedback mechanisms due to light scattering at grain/air and crystalline/glass interfaces. The phenomenon of replica symmetry breaking was verified through statistical analysis of the RLs intensity fluctuations, indicating a photonic phase-transition (corresponding to the RL threshold) analogous to the paramagnetic-to-spin glass transition in magnetic materials. The various results reported here highlight the potential of glasses and glass-ceramics for the development of RLs with improved performance in terms of reduction of laser threshold and large lifetime of the active media in comparison with organic materials.

## 1. Introduction

Random lasers (RLs) are optical systems that operate with basis on the multiple scattering of light propagating inside gain media to produce lasing without feedback by optical cavities [1,2,3,4,5,6,7]. Commonly used gain media include organic dyes, semiconductors, and dielectric nanocrystals doped with rare-earth ions (REI). The scattering elements, randomly spatially distributed inside the gain media, can be nanoparticles, porous structures, or disordered microstructures made of dielectric or metallic materials [6,7,8,9,10,11,12,13].

The choice of appropriate gain media influences the RL’s threshold and efficiency, as well as the emission characteristics, being a key decision for practical applications. For instance, the choice of different lasing materials can provide enlargement of the emission wavelength range, allowing studies across different spectral regions. The selection of appropriate materials may allow the development of compact RL devices [10,11,12,13,14,15,16,17,18,19,20].

The use of glasses and glass-ceramics (GCs) as hosts for RLs offers many benefits due to their specific physical properties, such as high optical damage threshold; the possibility to adjust their composition to modify the refractive index and optical absorption coefficients; incorporation of REI in the host material to obtain lasing in particular spectral regions; and engineered scattering centers inside bulk glasses and optical fibers to have control of some RL characteristics. Another possibility occurs when the system is composed of particles that act both as scatterers and as a gain medium, such as semiconductor nanoparticles, as well as amorphous and crystalline powders doped with REI. In the case of REI-doped glass fibers, optical feedback can be obtained, for example, due to light reflections in random Bragg gratings, written in fibers and rectangular waveguides [6,7,15,17,20].

In glasses, nanoscale density fluctuations are expected, due to the nature of the fabrication process and structure of these materials. The size, distribution, and contrast of these density domains are related to the glass composition and its thermal history and directly influence the light scattering efficiency and the effective mean free path of photons within the medium. When the characteristic dimensions of these heterogeneities approach or exceed the wavelength of light, multiple light scattering events become more prominent, enhancing the probability of localized optical feedback. Moreover, when glasses are ground into micrometric powders, additional scattering also occurs at the interfaces between the glass particles and the surrounding air. This external scattering mechanism dominates over intrinsic density fluctuations and ensures sufficient feedback for RL generation. In glass-ceramics, REI may preferentially incorporate into the crystalline phase, significantly modifying the RLs emission spectra and enhancing the coupling between emitters and scattering centers. This spatial overlap increases the probability that photons emitted by the dopant ions are efficiently scattered into regions containing other active ions, enhancing the likelihood of stimulated emission and thus promoting feedback and improving overall laser performance.

Glasses and GCs may be produced in various ways. Melt-quenching technique is the most common fabrication process for manufacturing glasses [21,22,23]. The raw materials must be previously well mixed and submitted to high temperatures to melt the mixture. The resulting liquid is then rapidly poured into a mold that must be colder than the transition temperature of the glass. Afterwards, the glass may be submitted to a posterior annealing treatment to reduce the thermal stresses resulting from the rapid cooling process. Glass powders are obtained by milling the glass until micrometric grains are obtained. Bulk glasses may also be modified by the irradiation with fs-lasers [24]. In this case, the refractive index of the glass may be locally modified, which enables the fabrication of optical devices, such as waveguides and optical amplifiers, for example. In addition, fs-irradiation of glass could also be used to control light scattering within the material, giving rise to novel optical devices. Amorphous thin films may also be obtained by RF-sputtering [25]. Thin films may be patterned, using lithographic techniques, that may also give rise to devices such as waveguides and optical amplifiers, among others [26]. In all the mentioned processes, the composition of the glass may be tuned to fine-adjust the properties of the resulting materials. Heat treatments may also be performed to carefully grow crystallites within the glass matrices, giving rise to glass-ceramics samples.

RLs based on glass and GCs powders doped with REI are not very frequently reported in the literature. Several years ago, an upconversion-RL (UC-RL) emitting in the ultraviolet (381 nm) based on a fluoroindate glass powder doped with neodymium ions (Nd^3+^) was reported [27]. The sample was pumped by a pulsed dye laser operating at 575 nm, in resonance with the Nd^3+^ transition ^4^I_9/2_ → ^2^G_7/2_. The RL threshold was 30 kW/cm^2^, and RL pulses of 29 ns were observed for pumping above the threshold. Considering the large variety of efficient UC processes involving REI-doped materials, the results showed the possibility to operate RLs with basis on other UC schemes [28].

Indeed, RLs in glass powders can be advantageous, since the broadband emission, caused by inhomogeneous broadening of the REI electronic transitions, can favor applications involving Q-switching and ultrafast lasers due to mode locking [29]. Furthermore, glasses have a simpler manufacturing process than crystals, and a large variety of glasses exhibit greater solubility limits for fluorophore ions. Works on RL in GCs are also rare in literature. However, GCs are interesting media for photonic devices, as they can withstand large pump power (up to 7 mJ/mm^2^ for germanate GCs) and have a high thermal damage threshold [30,31]. Moreover, GCs can be heavily doped with REI to alter their emission characteristics.

In this paper, we review recent works with RLs based on heavy-metal oxide glasses and GCs doped with REI. Tellurite and germanate glasses are strong candidates for the operation in RLs for several reasons. They present high solubility for REI, which is crucial for achieving high gains and minimizing the excitation energy threshold for laser operation. Although the solubility limits for the REI are not available in the literature, tellurite glasses containing up to 16 wt% of Nd_2_O_3_ and germanate glasses containing up to 10 wt% of Nd_2_O_3_ were reported [30,32]. It is important to add that although high REI concentrations may cause luminescence concentration quenching (LCQ) due to non-radiative energy transfer (ET) among REI [33,34,35], it does not affect RL performance since the laser emission occurs in a shorter time than the non-radiative ET. Indeed, in tellurite glasses, it was observed that high Nd^3+^ concentrations increase the quantum efficiency of the system, allowing it to reach the laser regime at low excitation energy.

These glasses also present large refractive index (~2.0), which is fundamental to improve light scattering that is necessary to provide feedback for RL operation. Additionally, these glasses present a wide transmittance window (400–5000 nm), which enables RL operation over a large spectral range, high nonlinear susceptibility, low phonon energy (~700 cm^−1^), and high resistance to optical damage.

The optical properties of tellurite and germanate glasses doped with REI were also extensively explored in the literature [21,22,31,36,37]. Conventional lasing in zinc-tellurite bulk glasses was already reported several years ago [21]. Optical gain was also achieved in tellurite and germanate glass and thin film-based waveguides doped with REI [24,26]. It is also worth mentioning that the composition of the glass plays a crucial role in its optical properties. The addition of alumina (Al_2_O_3_) in Nd-doped lead-germanate glasses (GPA glass) changes the symmetry of the Nd^3+^ environment, which favors the enhancement of the REI emission cross-section, resulting in an increase in the PL intensity at ~1064 nm. On the other hand, the addition of MgO to the composition of GP glass (GPM glass) contributes not only as a glass modifier, but also as devitrifying agent, contributing to the GC formation by GP glass devitrification [31,38]. Infrared-to-visible (green and red) UC was also extensively exploited in different germanate glasses and GCs doped with Er^3+^. Yb^3+^ is commonly added to the glasses’ compositions because the optical absorption cross-section of Yb^3+^ at 980 nm is much larger than the absorption cross-section of Er^3+^ and in such cases, Yb^3+^ acts as energy donor for the Er^3+^ [39]. This process is also discussed in the following sections in the context of RLs.

This paper is structured as follows: Section 2 describes the experimental procedures used for fabrication of tellurite- and germanate-based glasses and glass-ceramics. Conventional optical characterization techniques and a brief review of the procedures used for statistical study of emission intensity fluctuations are also presented in Section 2. Section 3 presents the results for Nd³⁺-doped glass systems, including TeO_2_-ZnO (TZO), TeO_2_-ZnO-Al_2_O_3_ (TZA), and GeO_2_-PbO-Al_2_O_3_ (GPA). Section 4 focuses on glass-ceramics based on the GeO_2_-PbO-MgO (GPM) system, doped with either Nd³⁺ or Er³⁺/Yb³⁺ pairs. Finally, Section 5 summarizes the main results. The discussion emphasizes the impact of glass composition, crystallization, and REI distribution on the light scattering properties and RL performance of the materials.

## 2. Experimental Details

### 2.1. Fabrication of Tellurite and Germanate Glass and Glass-Ceramics

The synthesis of tellurite and germanate glasses reported in this paper was based on the conventional melt-quenching technique. For the experiments, tellurite glasses were prepared by melting TeO_2_ with network modifiers, such as ZnO or Al_2_O_3_, and small amounts of Nd_2_O_3_ ([0.5, 1.0 … 10.0] wt.%) at temperatures of ~800 °C inside a platinum crucible for about 20 min. The melt was rapidly quenched in a preheated brass mold to prevent crystallization and to form a glassy state. Then, annealing was performed at 325 °C for 2 h and cooled down for 2 h to avoid internal stress. It is also possible to obtain glass powders by quenching the molten glass directly into water, at room temperature. This process facilitates the milling of the glass into a fine powder and helps to prevent undesirable crystallization of the glass. The germanate glasses were synthesized in a similar way, using GeO_2_ as the primary network former, also combined with oxides [PbO, Al_2_O_3_, MgO] to modify the glass structure. These glasses require melting at ~1200 °C, followed by rapid cooling.

The preparation of GCs from the germanate glasses follows a two-step process: (i) glass synthesis as described above followed by (ii) controlled crystallization that consists of a heat treatment near the glass transition temperature to favor nucleation and crystallite growth. The heat-treatment temperature is kept near the crystallization onset of the glass, obtained from Differential Scanning Calorimetry (DSC) measurements (approximately 830 °C for germanate glasses) during a period that may vary from 0.5 h to 5 h while maintaining transparency. The duration and temperature of the heat treatment may vary for different doping levels and glass matrix compositions and determine the crystal phases, crystallite average sizes, and optical properties of the GC.

The approach described above allowed formation of composite materials with both amorphous and crystalline phases, with enhanced optical nonlinearity, large mechanical strength, and good thermal stability. Finally, the samples were analyzed using X-ray diffraction (XRD) to confirm crystallinity, scanning electron microscopy (SEM) for microstructure evaluation, and optical spectroscopy to assess REI emissions.

More details on the sample’s fabrication are given in references [30,32,33,39,40].

### 2.2. Optical Characterization of the Samples

**Figure 1:**
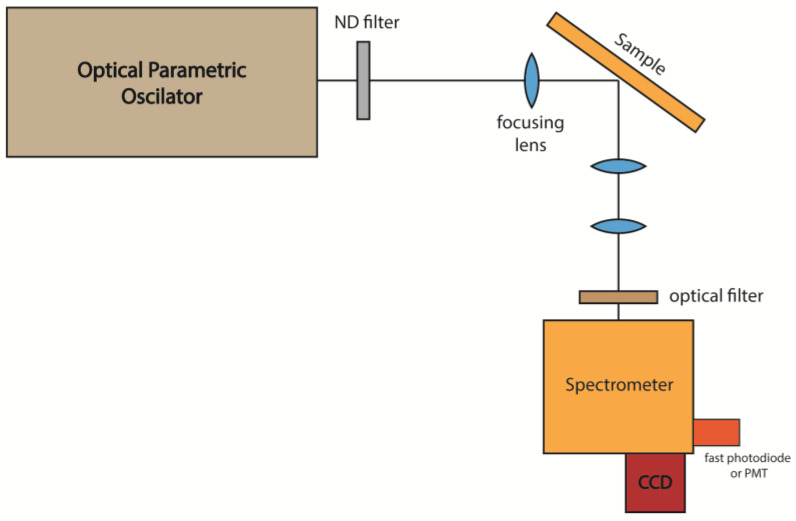
Typical setup for RL measurements in glass and GCs powders.

The optical characterization of the samples was conducted to evaluate their potential for RL operation. The excitation source consisted of a pulsed Optical Parametric Oscillator (OPO), delivering (pulses of ~7 ns) at tunable wavelengths, selected according to the absorption characteristics of the REI under investigation. The excitation wavelength was adjusted based on the dopant (e.g., Nd³⁺ or Er³⁺/Yb³⁺) and the specific emission transition being studied. The light emitted from the samples was directed to a high-resolution spectrometer that could be externally triggered, enabling the acquisition of emission spectra corresponding to individual excitation pulses. A typical experimental setup is illustrated in Figure 1.

The emission spectra were recorded as a function of the excitation pulse energy to evaluate the presence of stimulated emission and to determine the RL threshold. RLs based on glasses are not expected to present large narrowing of the emission line above the laser threshold as in RLs based on crystalline media because the optical transitions of the embedded laser-active ions are always affected by inhomogeneous broadening [34,35]. This is a result of the complex structure of the glassy hosts, where active ions may occupy several distinct sites with dissimilar chemical environments, resulting in many possible lasing modes. For this reason, even conventional lasers based on non-crystalline hosts may present wider emission bandwidths (Δλ>15 nm) when compared to crystalline hosts (Δλ<1 nm). Nevertheless, the RLs based on glasses still present a nonlinear input–output curve characterized by a laser threshold, and the photoluminescence (PL) dynamics are modified above the laser threshold.

Another way of verifying RL behavior is through temporal measurements where it is possible to see laser action through the appearance of a shorter peak on top of the longer PL decay [41]. The smallest pump fluence at which this peak is observed corresponds to the laser threshold.

In addition to the basic optical characterizations of the RLs, the statistical study of emission intensity fluctuations was also performed in some of the reviewed works. This characterization was motivated by several works [6,42,43,44] demonstrating that the RL transition is a photonic phase-transition, analogous to the paramagnetic-to-spin glass phase-transition in magnetic materials. The phase-transition is characterized by the replica symmetry breaking (RSB), a concept introduced by G. Parisi in the context of magnetic spin-glass theory [45,46]. Replicas, in the RL context, are the output spectra of the RL system observed in a time-interval, obtained under identical experimental conditions. The search for RSB is based on the analogies between (i) the magnetic spins and the optical modes observed in the emission spectra of the RL, and (ii) the inverse temperature the magnetic system and the laser pump intensity. The probability density function, $P\left(q_{\alpha \beta }\right)$, measures the degree of correlation among the spectral modes being associated with the Parisi overlap parameter in the spin-glass theory, $q_{\alpha \beta }$, [45,46] which measures the correlation between intensity fluctuations in different experimental replicas. For example, in the case of pulsed lasers, a replica corresponds to the spectrum emitted for each laser pulse and $q_{\alpha \beta }$ is defined as [42,43,44,45,46,47,48].

(1) $$q_{\alpha \beta }=\frac{\sum_{i=1}^{N}{∆}_{\alpha }(i){∆}_{\beta }(i)}{\sqrt{[\sum_{i=1}^{N}{∆}_{\alpha }^{2}\left(i\right)][\sum_{i=1}^{N}{∆}_{\beta }^{2}(i)]}}$$

where α,β=1,2,…,N are the replica labels. The intensity fluctuations are represented by ${∆}_{\alpha }\left(i\right)=I_{\alpha }\left(i\right)-\langle I\rangle (i)$ where $\langle I\rangle \left(i\right)$ is the average intensity at the wavelength $\lambda_{i}$ being determined by $\langle I\rangle \left(i\right)=\frac{1}{N}\sum_{\alpha =1}^{N}I_{\alpha }\left(i\right).$Another way used to characterize the RL action was the use of Pearson correlation coefficient (PCC) maps [43] which measure the correlations between the optical modes emitted in each replica. The PCC, $C_{ij}$, can be calculated as follows [43]:

(2) $$C_{ij}=\frac{\sum_{\alpha =1}^{N}{∆}_{\alpha }(i){∆}_{\alpha }(j)}{\sqrt{[\sum_{\alpha =1}^{N}{∆}_{\alpha }^{2}\left(i\right)][\sum_{\alpha =1}^{N}{∆}_{\alpha }^{2}(j)]}}$$

The coefficient $C_{ij}=0\;$indicates that the modes $\lambda_{i}$ and $\lambda_{j}$ are statistically uncorrelated. A positive (negative) $C_{ij}$ indicate that $\lambda_{i}$ and $\lambda_{j}$ are positively (negatively) correlated, indicating that they are spatially overlapped modes and compete for gain.

Ghofraniha et al. [44] observed RSB in a RL, for the first time, by performing a statistical investigation of the intensity fluctuations of the spectral modes below and above the RL threshold. The spectral modes correspond to the wavelengths emitted by the system under a certain excitation intensity. Below the RL threshold, the optical modes are uncorrelated, giving rise to a symmetrical replica distribution $P\left(q_{\alpha \beta }\right)$ with the Parisi overlap parameter centered at q=0 . However, above the RL threshold, the modes are highly correlated, and the replica symmetry is broken, shifting the maximum values of $P\left(q_{\alpha \beta }\right)$ for q = ±1. This photonic-phase-transition from spontaneous emission regime to the photonic spin-glass analogue was already demonstrated theoretically and experimentally observed for all systems cited above [42,43,44,45,46,47,48].

**Figure 2:**
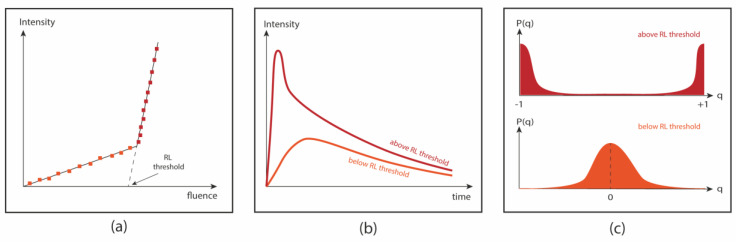
Schematics summarizing the features to characterize RL in glasses and GCs: (**a**) Input–output nonlinear curve showing the RL threshold; (**b**) typical temporal dynamics below and above RL threshold; (**c**) plot of the probability density function of the Parisi parameter $P\left(q\right)\;as\;a\;function\;of\;q$. Below threshold, *P*(*q*) is centered at q=0, indicating symmetrical replica distribution. Above threshold, the replica symmetry is broken and the value of $q_{max}$ shifts to ±1.

Figure 2 summarizes the main characterization features for RL action in glass and GCs.

## 3. Glass-Based Random Lasers

### 3.1. Nd ^3+^-Doped TeO_2_-ZnO (TZO)

Among the possible non-crystalline candidates for RLs media, tellurite glasses present numerous attractive properties, such as a large refractive index (~2.0), high optical nonlinear susceptibility, high chemical stability, and low-phonon energy (~700 cm^−1^) [35]. Indeed, several previous works regarding the investigation of REI-doped tellurite glasses are available in the literature. Previous reports of TeO_2_-ZnO glass demonstrated applications for conventional lasers [21], memory devices [25], enhancement of UC process [22], optical thermometers [23], and white light generation [49].

**Figure 3:**
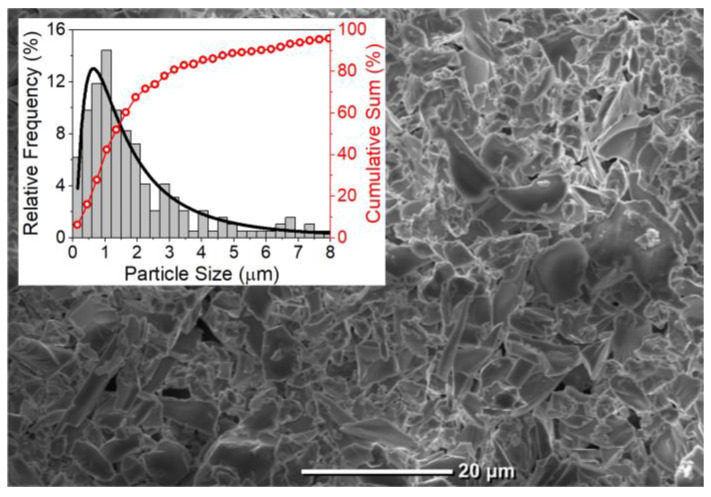
SEM image of the milled Nd^3+^-doped TZO glass. The inset shows the histogram of the size distribution of the particles fitted with a log-normal curve (in black) and the percentage of cumulative sum of sizes (in red).

The first RL based on tellurite glasses was reported in 2021 [40] and is reviewed in this section. Figure 3 shows a SEM image of the sample, which is predominantly composed of particles with an average size of 1 µm.

**Figure 4:**
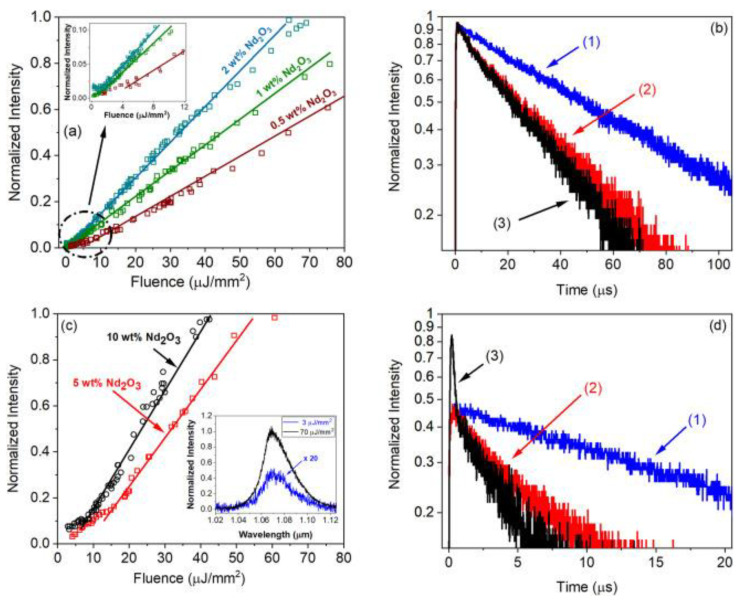
Emitted intensity vs EFE curves and temporal behavior of the signal (at ~1068 nm) for Nd^3+^-doped TZO glass-powders: (**a**) illustrates the curves of emitted intensity vs EFE for samples doped with 0.5, 1.0 e 2.0 wt. % of Nd_2_O_3_; (**b**) temporal behavior for the sample doped with 1.0 wt. % of Nd_2_O_3_; (**c**) intensity vs EFE for samples doped with 5.0 and 10.0 wt. % of Nd_2_O_3_; the inset shows the corresponding emission curves for the sample doped with 10.0 wt. % of Nd_2_O_3_ for EPE below and above threshold; (**d**) illustrates the temporal behavior for the sample doped with 10.0 wt.% of Nd_2_O_3_; (1) 808 nm diode laser pumping; (2) OPO pumping at 585 nm with 3 μJ/mm^2^; and (3) OPO pumping at 585 nm with 100 μJ/mm^2^. Adapted from [40], Copyright (2025), with permission from Elsevier.

The RL characterization in the TZO glass was carried out evaluating the emitted intensity as a function of the excitation fluence and the temporal response for excitation below and above threshold. Figure 4a shows the results of the emitted intensity at 1068 nm versus the Energy Fluence Excitation (EFE) for samples doped with small Nd^3+^ concentrations. For low pumping energies (below 4 μJ/mm^2^), a smooth transition between two distinct PL regimes can be seen. However, the emission decay time was gradually reduced as the EFE increases for the sample doped with 1.0 wt. % of Nd_2_O_3_, as presented in Figure 4b. The PL temporal decay reduced from 140 μs, when the sample was pumped by a low power diode laser, to 48.2 and 40.6 μs, when excited with 3 and 100 μJ/mm^2^ by a pulsed laser beam, respectively. The rise of population inversion explains this behavior by increasing the density of stimulated photons.

Two different regimes can be seen in Figure 4c, for samples doped with 5.0 and 10.0 wt.% of Nd_2_O_3_, respectively, characterized by energy fluence thresholds at 8 and 5 μJ/mm^2^; the inset shows the results of the corresponding emissions for the sample doped with 10.0 wt.% for EFE below and above threshold. For pumping above the RL threshold at 70 µJ/mm^2^, the emission intensity at 1068 nm was ~20 times higher in comparison to the sample that was pumped at 3 µJ/mm^2^, below RL threshold. We highlight that for excitation energies above thresholds, a fast emission decay in the nanosecond, super imposed on a signal in the μs range, is observed for the sample doped with 10.0 wt.% of Nd_2_O_3_, as presented in Figure 4d. This reduction is in accordance with the expected RL dynamics that follow the excitation pulse temporal profile.

### 3.2. Nd ^3+^-Doped TeO_2_-ZnO-Al_2_O_3_ (TZA)

RL emission at 1337 nm was recently observed in TeO_2_-ZnO-Al_2_O_3_ (TZA) glass samples doped with three concentrations of Nd_2_O_3_ (4, 8, and 16 wt.%) by monitoring the emission dynamics and the output power [32]. The experiments were motivated by previous PL results that showed the influence of the Al_2_O_3_ addition in the glass composition, which enhanced the PL intensity in Nd^3+^-doped TeO_2_-ZnO glasses.

**Figure 5:**
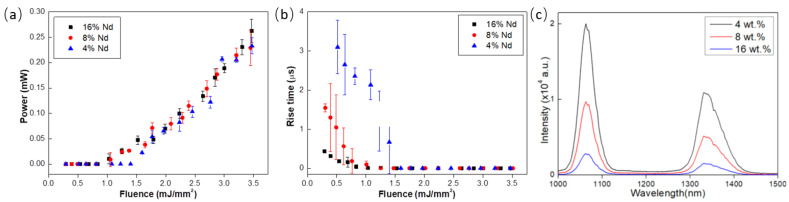
(**a**) Maximum peak power per fluence. (**b**) Rise time measurements for samples with 4 wt.% (blue triangles), 8 wt.% (red circles), and 16 wt.% (black squares) Nd_2_O_3_ doping. (**c**) Emission spectra of the samples. Figure 5c was Reprinted from [32].

Figure 5a,b show a clear threshold that coincided to within 15% with the change in slope of the rise time measurements. For the lowest Nd_2_O_3_ concentration (4 wt.%), the highest threshold was observed at (1.4 ± 0.4) mJ/mm^2^, whereas the sample with 16 wt.% Nd_2_O_3_ demonstrated the smallest threshold (0.81 ± 0.10) mJ/mm^2^.

The emission at 1337 nm was observed for the first time in an RL, whereas no laser emission at 1064 nm was detected. This behavior was attributed to the temperature growth within the thin active laser layer, which might have favored the population of the ^4^I_11/2_ energy level, hampering population inversion that would generate emission at 1064 nm.

**Figure 6:**
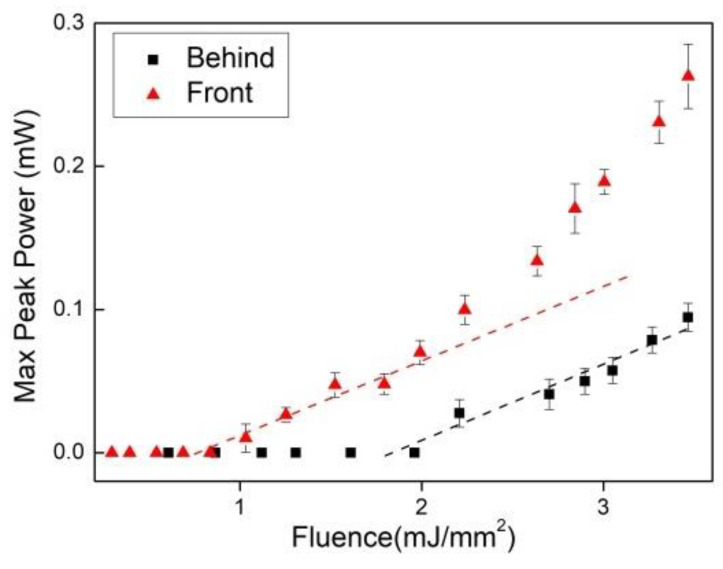
Maximum peak power per EFE for the 16 wt.% Nd_2_O_3_ sample for pumping in the powder surface from the front (red triangles) and for pumping from behind through the glass slide (black squares). Reprinted from reference [32].

A large decrease in laser intensity was observed when pumping the sample from the back through the glass slide and when performing the measurement parallel to the sample surface (Figure 6). A reflection reduction at the index-matched glass-sample interface and cavity geometry, respectively, may be the reason that explains and corroborates our interpretation of a coherent laser emission originating inside the cavity formed by the sample surface and the gain–loss boundary that took place inside the sample. This mechanism was pronounced and became stronger as the mean-free-path of transport approached the macroscopic absorption length, which, in the present case, occurred with the absorption increase, i.e., for the higher wt.% Nd_2_O_3_ sample.

### 3.3. Nd^3+^ Doped GeO_2_-PbO-Al_2_O_3_ (GPA)

Germanate glasses represent another class of materials that have been widely studied through PL experiments. For example, glassy hosts based on GeO_2_ and doped with REI demonstrated applications for white light generation [37], photovoltaic devices [36], waveguides produced with fs laser irradiation [24], and Si technology for operation at the near-infrared region, among others [26].

Particularly, lead-germanate (GP) glasses are strong candidates for the operation of RLs, as they have large solubility for REI, large refractive index (~2.0), wide transmittance window (400–5000 nm), high nonlinear susceptibility, and high resistance to optical damage. Recently, large PL growth in the near infrared (~1064 nm) was observed in GP glasses by addition of alumina in their composition (GPA glasses), since Al_2_O_3_ changes the symmetry of the coordination environment of Nd^3+^ ions [32]. This result motivated the investigation reviewed in this Section.

**Figure 7:**
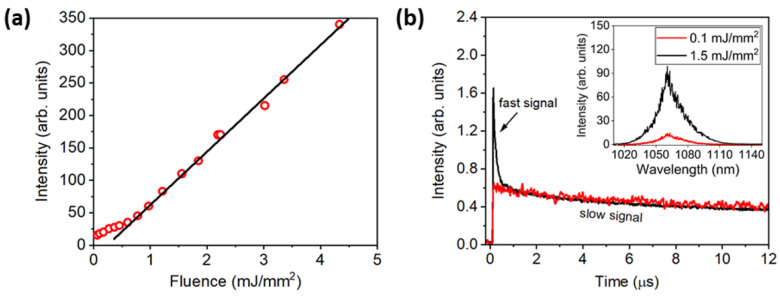
(**a**) Emitted intensity at 1060 nm versus the EFE. (**b**) Signal at 1060 nm: temporal decay for EFE < (EFE)_th_ (red line) and for EFE > (EFE)_th_ (black line) (the *inset* shows the corresponding PL spectra for EFE below and above the RL threshold). Excitation at 808 nm (^4^I_9/2_ → {^4^F_5/2_, ^2^H_9/2_}). Reprinted with permission from [33] © Optica Publishing Group.

Figure 7a illustrates the emitted intensity at 1060 nm versus EFE at 808 nm (^4^I_9/2_→{^4^F_5/2_, ^2^H_9/2_}) for Nd^3+^-GPA powder; we observe clearly the transition from spontaneous to RL emission at about 0.3 mJ/mm^2^. Moreover, for EFE below and above the RL threshold (EFE)_th_ (*inset* of Figure 7b), an intense PL band centered at approximately 1060 nm was noticed (due to the transition ^4^F_3/2_→^4^I_11/2_). As expected, due to the inhomogeneous broadening of the electronic transition, both for EFE smaller and larger than (EFE_th_), the full width at half maximum (FWHM) of the PL band is ~30 nm.

Further, Figure 7b shows the temporal behavior of the emission centered at 1060 nm in which only the spontaneous emission with decay time of 10 μs (red curve) is present below the RL threshold of about 0.1 mJ/mm^2^. For a similar GPA glass containing only 0.5 wt.% of Nd_2_O_3_, the decay time is around 200 µs. The ET mechanisms due to LCQ favor non-radiative relaxations from the ^4^F_3/2_ energy level to lower Nd^3+^ energy levels [35] and explain the decay time reduction. The large energy gap between ^4^I_13/2_ and ^4^I_15/2_ energy levels indicates that multiphonon relaxation is not relevant in this case [35]. However, amplified spontaneous emission (ASE) is occurring even at low values of EFE, leading to a short decay time, due to the rise of population inversion that leads to an increase of the density of stimulated photons. Therefore, the output emission presents a fast signal of about 10 ns, superimposed to the signal of 10 μs due to stimulated emission and consistent with the expected RL dynamics due to the excitation laser pulses with 10 ns duration [41].

## 4. Glass-Ceramics-Based Random Lasers

### 4.1. Nd^3^-Doped GeO_2_-PbO-MgO (GPM)

When MgO is included in the composition of GP glass (forming GPM glass) it acts as a glass modifier and as a devitrifying agent, contributing to GCs formation by GP glass devitrification [30]. We review in this section the studies related to RL operation in Nd^3+^-doped GPM GCs, reported for the first time in [30].

The reagents were melted at 1200 °C in a platinum crucible for 1h, and then quenched in water at room temperature to prevent crystallization. The resulting GPM glasses were ground using a mortar and pestle to obtain a fine powder. Approximately 18 mg of the GPM powder were submitted to Differential Scanning Calorimetry (DSC) analysis (model Labsys Evo, manufactured by Setaram at Caluire-et-Cuire, France), to verify the most suitable temperatures for the crystallization process. DSC analysis was conducted in N_2_ atmosphere (100 mL/min) using an alumina crucible and heating rate of 20 °C/min.

The GPM powder was separated in five samples labelled GPM, GPM05, GPM1, GPM3, and GPM5. Each sample was submitted to devitrifying heat treatments at 830 °C, in air, for different times [GPM05-0.5 h; GPM1-1.0 h; GPM3-3.0 h; GPM5-5.0 h].

**Figure 8:**
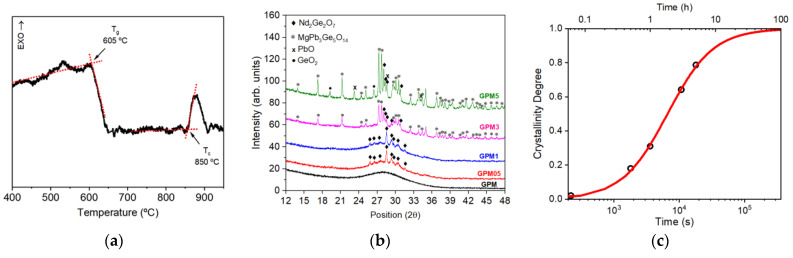
(**a**) DSC of GPM (10 wt% of Nd_2_O_3_) glass sample at heating rate of 10 °C/min (Tg = glass transition temperature, Tc = crystallization temperature). (**b**) X-ray diffraction for Nd^3+^-doped samples GPMx (x = 0.5, 1, 3, 5). (**c**) Crystallinity degree as a function of annealing time. Reprinted from reference [30].

The annealing temperature was slightly below the onset of crystallization that was determined from DSC measurements (Figure 8a). After the heat treatment, each GC sample was subjected to a second pulverization process. Then, the obtained powders were submitted to X-Ray diffraction (XRD) in a Rigaku SmartLab advanced X-ray diffractometer (manufactured by Rigaku, at Tokyo, Japan) with CuK_α_ radiation ($\lambda_{x}=0.154059\;nm;\;40\;kV;\;30\;mA$) to follow the structural changes of the specimens (step size: 0.01°; time per step: 0.3 s), which is shown in Figure 8b. The average size (D) of crystallites was estimated from the width of X-ray peaks according to Scherrer’s equation $D=\frac{K\lambda_{x}}{\beta \cos\theta }$, where $\lambda_{x}$ is the wavelength of X-ray radiation, θ the diffraction angle, β is the width of peak at half of its maximum intensity (FWHM), and K is a dimensionless shape factor known as the Scherrer constant (in the case of spherical particles K=0.94). The percent crystallinity degree (CD), shown in Figure 8c, was estimated by the ratio of the crystalline area, AC, present in the diffractogram of the devitrified glass (glass-ceramics) and the total area, AT (amorphous + crystalline), using the equation $D=100\frac{AC}{AT}$.

We observed and characterized in the present case the photonic analogue behavior of the paramagnetic-to-spin glass phase transition in magnetic materials. The change from the spontaneous emission regime (below the laser threshold) to the RL glassy behavior (above the threshold) was characterized as described in Section 2.2.

The Parisi overlap parameter $q_{\alpha \beta }$ of Equation (1) was calculated from N=200 acquired PL spectra and obtained for each EFE used to analyze the RL modes correlations. Then, the probability distribution P(q)*,* $q=q_{\alpha \beta }$ was determined for all samples and for various EFEs.

**Figure 9:**
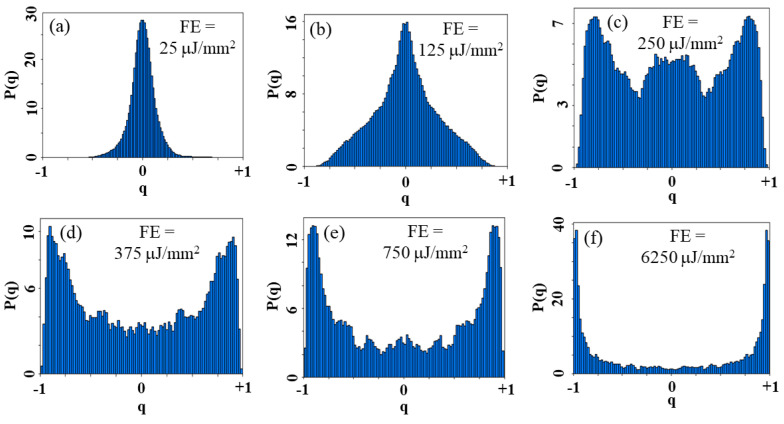
Probability distribution of the Parisi overlap parameter for different EFEs. Below threshold: (**a**) EFE = 25 μJ/mm^2^, and (**b**) EFE = 125 μJ/mm^2^. In both cases the maximum of P(q) occurs at q≅0. (**c**) On the threshold: EFE = 250 μJ/mm^2^; the maximum of P(q) occurs at q≅±1. Above the RL threshold, (**d**) EFE = 375 μJ/mm^2^, (**e**) EFE = 750 μJ/mm^2^, and (**f**) EFE = 6250 μJ/mm^2^; the maximum of P(q) occurs at q≅±1. Reprinted from reference [30].

Figure 9 shows the behavior of P(q) for the GPM5 sample. Figure 9a shows that $q_{max}≅0$ when EFE < (EFE)_th_ indicating absence of correlations among the optical modes. Figure 9b–f show the behavior of P(q) as EFE is increased. When EFE ≅ (EFE)_th_, $q_{max}$ shifts to near −1 and +1, which indicates correlated and anti-correlated fluctuations, respectively. The large magnitude of P(q=0) in Figure 9c indicates that a significant number of replicas are not correlated. As presented in Figure 9d–f, RSB takes place for EFE > (EFE)_th_ and the maximum of P(q) consolidates at ${|q}_{max}|≅1$ for EFE >> (EFE)_th_.

We recall that the RSB phenomenon was reported for various RLs systems [6,42,44,47,48] but reference [30] reports the first time that it was observed for a RL based on a GC.

**Figure 10:**
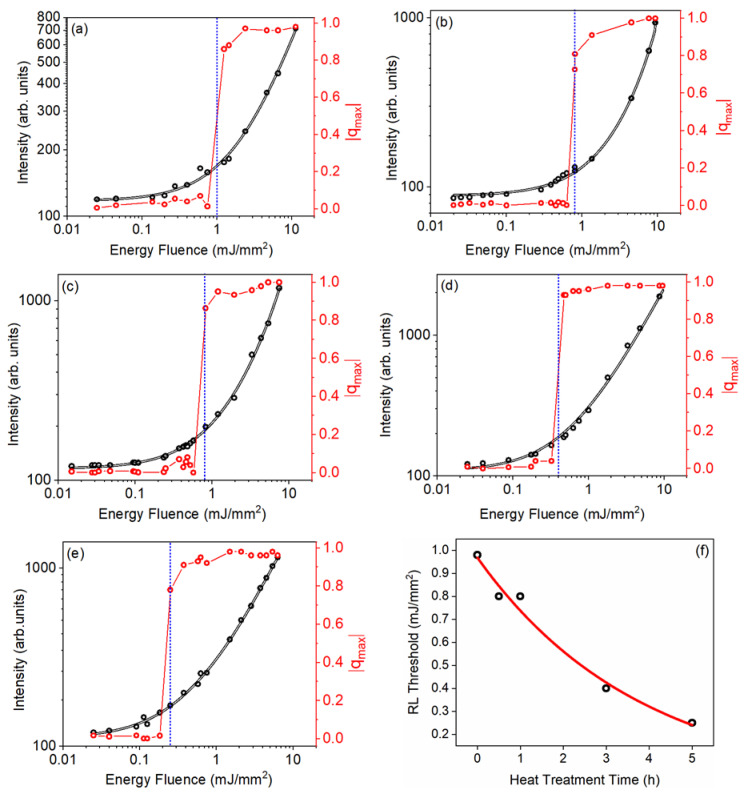
Modulus of the maximum Parisi overlap parameter (red curves) and PL intensity versus EFE at 1068 nm (black curves) for Nd^3+^-doped GPM samples. (**a**) GPM; (**b**) GPM05; (**c**) GPM1; (**d**) GPM3; (**e**) GPM5; (**f**) (EFE)_th_ as a function of heat treatment time. The dotted blue line indicates the (EFE)_th_ for each sample. Observe that (EFE)_th_ is reduced from ~1 to ~0.25 mJ/mm when we compare the untreated glassy GPM sample with the one submitted to the crystallization treatment for 5 h. The results of (EFE)_th_ as a function of annealing time in Figure 10f show clearly that the degree of crystallization is also correlated to the RL performance. Reprinted from reference [30].

The PL intensity at 1068 nm (black lines) and ${|q}_{max}|$ (red lines) versus the EFE for all samples is presented in Figure 10. Notice that the (EFE)_th_ obtained from the PL vs EFE curves agrees with the (EFE)_th_ determined from the ${|q}_{max}|$ curves. This is the normal behavior observed for other already reported RLs [6,42,44,47,48].

**Figure 11:**
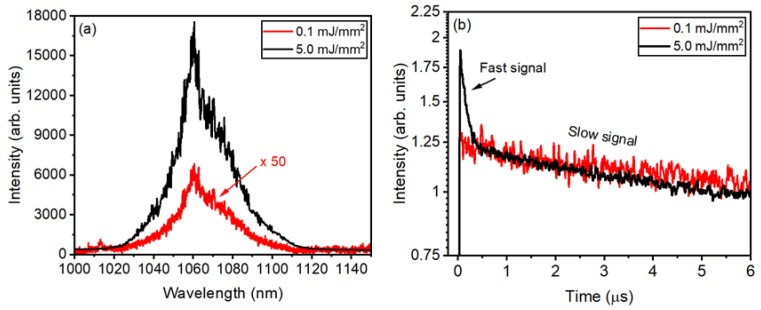
(**a**) Emission spectrum and (**b**) temporal behavior of the signal (at ~1068 nm) for the GPM5 sample for EFE below and above (EFE)_th_. Adapted from reference [30].

Figure 11a shows that the PL intensity at 1068 nm becomes about 130 times larger when the EFE is increased from 0.1 to 5.0 mJ/mm^2^ for the GPM5 sample. However, any significant reduction in the width of the emission band was observed. Figure 11b illustrates the PL dynamics below and above the (EFE)_th_ for the GPM5 sample. Below (EFE)_th_, the signal decay time was in the μs range for all samples. For EFE > (EFE)_th_, a fast signal was observed, in the nanosecond range superimposed on the slower signal (in the μs range). The slow signal is due to the spontaneous emission by the ions that are not participating in the stimulated emission process. The temporal behavior observed from the other samples is like the one displayed in Figure 11b.

The optical feedback mechanism in the RL based on the GPM sample is attributed to the multiple light scattering by the powder grains. For the GCs samples [GPM0.5; GPM1.0; GPM3.0; and GPM5.0], crystallites grown due to the heat treatment may also act as scatterers. This additional light scattering mechanism may increase the photon residence time within the grains contributing for reduction of the RL threshold. Moreover, the Nd^3+^ inside the crystallites grown within the glass phase may present sharper emission lines and may not suffer from large inhomogeneous broadening. Although a reduction on the emission linewidth above (EFE)_th_ was not observed, the RL slope-efficiency increased for the samples with a higher crystalline degree. Therefore, despite the inhomogeneous broadening caused by the residual glass, we expect that the crystallites act both as scatterers and as more efficient light emitters.

### 4.2. Er^3+^/Yb^3+^-Doped GeO_2_-PbO-MgO (GPM)

Infrared-to-visible UC has been exploited in different glasses and GCs doped with Er^3+^ by many groups. Normally, to improve the UC process, Yb^3+^ is added to the glasses’ compositions because its optical absorption cross-section at 980 nm is much larger than the absorption cross-section of Er^3+^; then, Yb^3+^ acts as energy donor to the Er^3+^ which emits green and red light. Therefore, to obtain an RL operating in the visible range, we performed RL experiments in Er^3+^/Yb^3+^ co-doped germanate–lead–magnesium (GPM) glass-ceramics [39].

**Figure 12:**
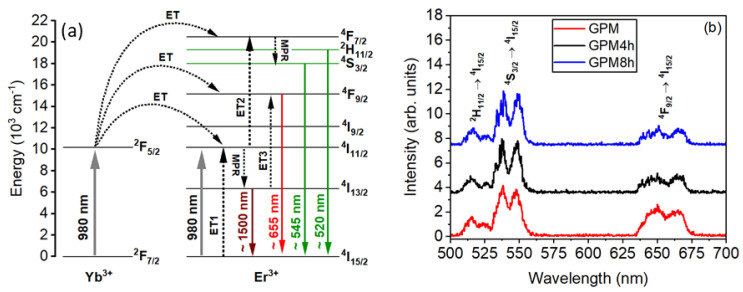
(**a**) Er^3+^/Yb^3+^ energy level diagram and the most important excitation process. (ET = Energy Transfer and MPR = Multi Phonon Relaxation). (**b**) Photoluminescence spectra for the Er^3+^/Yb^3+^ co-doped GPM glass-ceramics powders. Reprinted from reference [39], Copyright (2025), with permission from Elsevier.

Figure 12a shows the Er^3+^ and Yb^3+^ energy levels contributing to the experiments, and Figure 12b shows the PL spectra obtained from GPM, GPM4h, and GPM8h samples.

The UC-RL characterization was performed by measuring the laser threshold and spectrum, the overlap parameters $q_{\alpha \beta }$, and the correlation coefficients $C_{ij}$.

**Figure 13:**
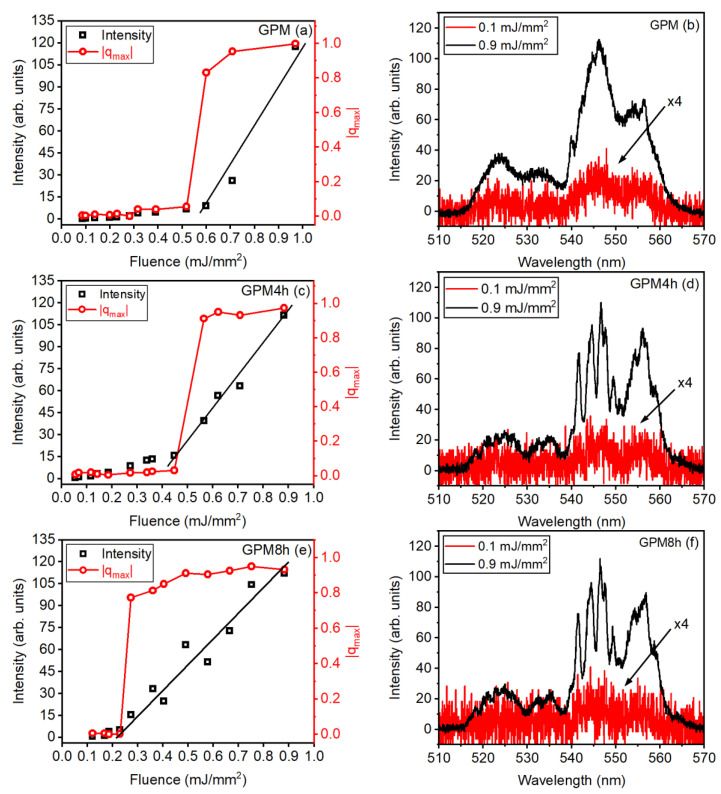
Modulus of the maximum Parisi overlap parameter $\left|q_{max}\right|$ (red curves) and PL intensity at 547 nm (black curves) versus EFE for samples: (**a**) GPM, (**c**) GPM4h, and (**e**) GPM8h. PL spectra for EFE below (red curves) and above (black curves) the (EFE)_th_ for samples (**b**) GPM, (**d**) GPM4h, and (**f**) GPM8h. Reprinted from reference [39], Copyright (2025), with permission from Elsevier.

Figure 13a,c,e shows the behavior of the emitted intensity at 547 nm *versus* the EFE for all samples, as well as the respective values of |$q_{max}|$, obtained from the most frequent values of *q* in the probability distribution *P(q*). A transition from the spontaneous emission regime to the RL regime is observed from the curves in Figure 13a,c,e. The RL threshold, (EFE)_th_, obtained from the PL curves agrees with the values found from the |$q_{max}|$ values for all samples. The transition of the |$q_{max}|$ values from 0 to 1 at (EFE)_th_ also reveals the occurrence of RSB, above (EFE)_th_.

The PL spectra for excitation below and above the (EFE)_th_ are shown in Figure 13b,d,f. The splitting of the spectra located between 540 nm and 550 nm, for the GC samples excited at 0.9 mJ/mm^2^, can be seen, indicating that a fraction of the ions contributing for the RL emission are located inside the crystallites.

Notice in Figure 13a,c,e that (EFE)_th_ is reduced from ~0.6 mJ/mm^2^ for sample GPM, and to ~ 0.45 and ~0.20 mJ/mm^2^ for samples GPM4h and GPM8h, respectively; this reduction indicates the influence on (EFE)_th_ of the samples’ crystallization degree. Indeed, the value of (EFE)_th_ is due to the multiple scattering of light at the particle–air interfaces in the powder, as well as among crystallites inside the particles; the change of the crystallites concentration modifies the photons mean-free-path and may lead to (EFE)_th_ reduction.

The samples were heat-treated for 4 and 8 h and presented sharp emission bands in the PL spectra. This indicates that a fraction of the REI originally in the samples diffused into the crystallites, where they can be in ErO_7_ or YbO_7_ polyhedral. This is validated by XRD results that demonstrated the presence of (Er,Yb)_2_Ge_2_O_7_ and (Er,Yb)_2_Pb_2_O_7_ phases within the samples, indicating that both the glass matrix and the small crystallites play the role of scatterers and as gain media.

Figure 13b,d,f present the PL spectrum for excitation below and above the (EFE)_th_. For the GC samples excited at 0.9 mJ/mm^2^, splitting of the spectra located between 540 nm and 550 nm is observed, indicating that a fraction of the ions contributing to the RL emission are located inside the crystallites.

**Figure 14:**
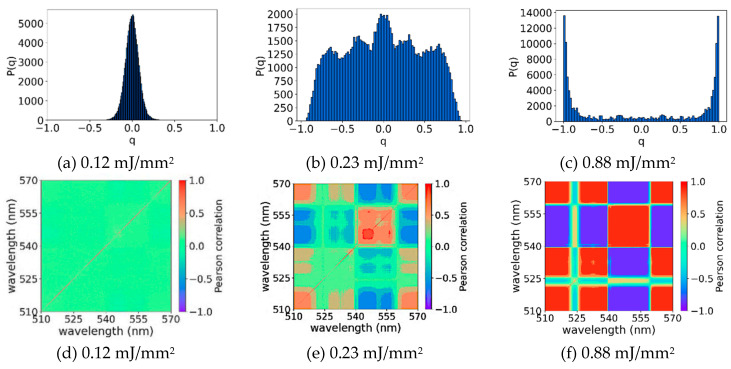
Probability distribution *P*(*q*) of the Parisi overlap parameter (top line) and respective Pearson correlation coefficients maps (bottom line) for sample GPM8h at the same experimental condition (same column) and at distinct excitation powers (different columns). Below the RL threshold: (**a**,**d**) EFE = 0.12 mJ/mm^2^. In this case, the maximum of *P*(*q*) occurs at $q_{max}≅0$ and the intensity fluctuations (Pearson correlation) are mostly uncorrelated ($C_{ij}=0)$. Near the RL threshold: (**b**,**e**) EFE = 0.23 mJ/mm^2^; peak profiles of P(q) start to appear towards the extremes $q_{max}≅\pm \;1$, indicating the beginning of the RSB behavior, and the intensity fluctuations presents correlation (red) and anti-correlation (blue) among distinct wavelengths of the emitted light. Red diagonals indicate self-correlation. Above the RL threshold: (**c**,**f**) EFE = 0.88 mJ/mm^2^; the maximum of *P(q)* consolidates at $q_{max}≅\pm \;1$ and the intensity fluctuations present modes competition for gain and presence of both correlation (red) and anti-correlation (blue) well-defined regions in the Pearson correlation maps. Reprinted from reference [39], Copyright (2025), with permission from Elsevier.

Figure 14a, obtained with EFE = 0.12 mJ/mm^2^, shows $q_{max}\approx 0$, which is consistent with the regime characterized by symmetric replicas expected for EFE < (EFE)_th_. This result is corroborated by the PCC calculations shown in Figure 14d. In this case, $C_{ij}$ is nearly zero for the whole map, which indicates absence of correlations among the output intensity fluctuations. The thin red line along the diagonal represents self-correlations. As the EFE increases, the shape of *P*(*q*) changes. In Figure 14b for EFE = 0.23 mJ/mm^2^, $q_{max}$ shifts toward −1 and +1, which indicates anti-correlated and correlated fluctuations, respectively. However, a significant number of replicas are not correlated, as indicated by the large magnitude of *P*(*q = 0*), a situation where EFE ≅ (EFE)_th_. In Figure 14e, corresponding to EFE = 0.23 mJ/mm^2^, we observe in the PCC map a clear correlation (red) and anti-correlation (blue) between the emitted modes that share or compete for gain in the nonlinear regime, indicating the beginning of the laser regime. For a higher EFE (0.88 mJ/mm^2^), a complete RSB glassy state occurs, and the maximum of *P*(*q*) is observed at $\left|q_{max}\right|≅1$, as shown in Figure 14c. The PCC map of Figure 14f exhibits well-defined red and blue regions that, respectively, denote correlation and anticorrelation regions of the emitted modes, because of the high competition for the available gain. Similar behavior was observed for the samples GPM and GPM4h. These results are evidence that the RL regime was achieved for all samples.

**Figure 15:**
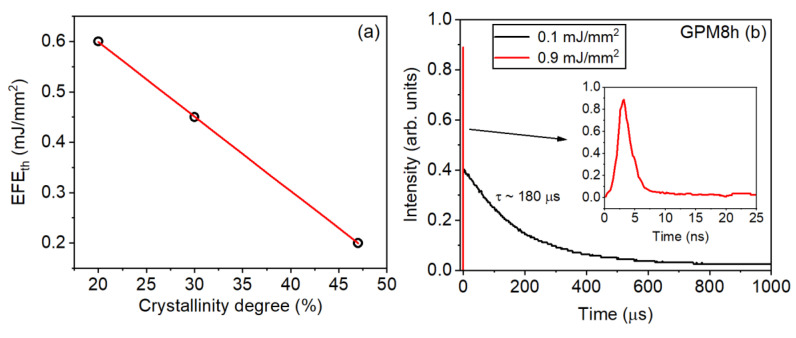
(**a**) Relationship between the (EFE)_th_ and the crystallinity degree. (**b**) PL dynamics below (black curve) and above (red curve) the (EFE)_th_ at 547 nm. Reprinted from reference [39], Copyright (2025), with permission from Elsevier.

The dependence of (EFE)_th_ with the degree of crystallinity is shown in Figure 15a, whereas in Figure 15b the PL dynamics at 547 nm, for excitation below (black line) and above (red line) the (EFE)_th_, for sample GPM8h, is presented. It is important to highlight that for EFE < (EFE)_th_ the emitted PL was in the µs range, which is consistent with the spontaneous emission regime. On the other hand, for $EFE=4.5\times (E{FE)}_{th}$, only a fast signal in the nanosecond range was observed, which is evidence of stimulated emission regime. Notice that in previous work [40], we showed that slightly above (EFE)_th_, both the fast and slow signals were observed for glass and GC samples. However, in the present case, the intensity of the PL signal for $EFE\approx (E{FE)}_{th}$ was smaller than the detection limit of the photodiode used. The PL dynamics results were similar for the samples GPM and GPM4h.

To conclude, we recall that the RSB was reported for various RLs based on liquids, crystals, and polymers [6,7,42,44], but the experiment reviewed in this section is the first example for a UC-RL based on the energy transfer among REI hosted in a glass-ceramic. Of course, since many glass compositions may be submitted to controlled crystallization, it may be feasible to obtain enhanced RL action in other glass-ceramics.

## 5. Summary and Conclusions

In summary, previous reports of RL action in tellurite and germanate glasses and glass-ceramics doped with rare-earth ions (REI) were reviewed and analyzed. The selection of host matrix and REI concentration strongly influenced RL performance. The optical feedback in the RLs was due to multiple light scattering at grain/air and crystal/glass interfaces. The RL emission was confirmed through nonlinear input–output curves and photoluminescence dynamics. Statistical analysis of the emission intensity fluctuations revealed a photonic-phase-transition characterized by replica symmetry breaking. The results indicate the possibilities for tuning RL properties through compositional modifications and crystallization control. Based on the experimental results, we expect that machine learning approaches may optimize glass compositions for enhanced RL performance, increasing the scientific and technological relevance of RLs based on glass and glass-ceramic for new photonic applications.

## Data Availability

No new data were created or analyzed in this review. Data sharing is not applicable to this article.
